# Why Is the Association Between Mediterranean Diet and Physical Performance in Athletes Inconclusive? Implications for Future Studies

**DOI:** 10.3390/jfmk10010016

**Published:** 2025-01-02

**Authors:** Alain Massart, Ádrian Rocha, José Pedro Ferreira, Carmen Soares, Maria João Campos, Diogo Martinho

**Affiliations:** 1 Faculty of Sport Sciences and Physical Education, University of Coimbra, 3040-248 Coimbra, Portugal; adriandelrocha@hotmail.com (Á.R.); jpl.ferreira.2010@gmail.com (J.P.F.); mjcampos@fcdef.uc.pt (M.J.C.); dvmartinho92@hotmail.com (D.M.); 2 Research Center for Sport and Physical Activity (CIDAF), 3040-248 Coimbra, Portugal; 3 Faculty of Arts and Humanities, Center for Classical and Humanistic Studies, University of Coimbra, 3004-530 Coimbra, Portugal; cilsoares@gmail.com

**Keywords:** nutrition, sports, gaps

## Abstract

**Background/Objectives**: Athletes increasingly turn to nutrition and supplements to enhance performance, yet the evidence surrounding the efficacy of the Mediterranean diet (MD) remains inconclusive. This scoping review aims to evaluate identify gaps in the existing literature and provide implications for future research on the MD’s potential to improve athletic performance across various demographics, sport types, and performance measures. **Methods**: We conducted a systematic search of peer-reviewed studies published across four databases (PubMed, Scopus, Scielo, and Google Scholar) using the following terms and their combinations: “mediterranean diet”, “performance”, “athlete”, “sport”. The search placed no restrictions on the experimental design or the time period of the publication and focused on athletes regardless of competitive level. We examined targeted studies about the MD’s effects on key performance metrics, such as endurance, strength, and speed, while also evaluating possible confounding factors like dietary intake, body composition, and training status. We further aimed to identify gaps in the literature by investigating the consistency of dosing protocols, supplementation duration, and methodologies used. **Results**: The preliminary findings suggest that while some studies report benefits of the MD for recovery and endurance, only 40% show statistically significant improvements in performance outcomes, often with methodological limitations. The studies also lacked homogeneity in participant demographics, dosage, and performance assessments, hindering comparability. Our review highlights the need for future research that incorporates standardized dosing, homogeneous athlete populations, and controlled dietary conditions. **Conclusions**: This review provides a framework to guide further research and offers insights into the potential role of the MD in sports nutrition.

## 1. Introduction

The Mediterranean diet (MD) emphasizes extra virgin olive oil as the main source of fat. It promotes the high consumption of fruits, vegetables, herbs, legumes, nuts, olives, seeds, spices, and whole grain cereals, with an emphasis on seasonal and fresh products. Moderate intake includes eggs, fish or seafood, low-fat dairy products, red wine, and white meat. It also implies limited consumption of red meat, processed foods, saturated fats, and sweets, such as sugary beverages, pastries, and other high-sugar foods [1,2,3,4].

The Mediterranean diet is a rich source of vitamins A, C, and D, as well as polyphenols and polyunsaturated fatty acids (PUFAs), which are well-known antioxidants. These components have been linked to reduced inflammation, a lower risk of infections, enhanced viral clearance, and improvements in the gut microbiome [5,6,7,8].

The MD plays a beneficial role in cardiovascular diseases (CVDs), diabetes, cancer, Alzheimer’s disease, and metabolic syndrome (including obesity, hypertension, hyperglycemia, and hyperlipidemia). Additionally, it serves as a protective factor against COVID-19, reduces overall mortality, improves cognitive function, attenuates post-traumatic stress disorder, has the potential to reduce healthcare costs, and promotes sustainability [3,4,5,6,7,8,9].

Adherence to the Mediterranean diet in conjunction with recreational exercise guidelines may offer a more comprehensive approach to achieving enhanced health benefits, in addition to those derived from the Mediterranean diet and exercise alone [4].

Regarding the potential benefits of the MD for physical performance in athletes, injuries or great charges of exercise can lead to cellular damage, inducing inflammation and oxidative stress, with negative effects on athletic performance or recuperation [10,11,12]. The MD includes a wide variety of antioxidant molecules that lead to significantly reduced reactive oxygen species (ROS) levels and to increased antioxidant defense (superoxide dismutase, catalase, and xanthine oxidase) and ROS detoxification [13]. The MD also has the potential to decrease inflammatory biomarkers, including tumor necrosis factor-alpha (TNF-α), interleukins (IL-1 and IL-6), C-reactive protein (CRP), cell adhesion molecule-1 (CAM-1), intercellular adhesion molecule-1 (ICAM-1), cyclooxygenase enzymes (COX-1 and COX-2), and nuclear transcription factor kappa B (NF-κB); additionally, it may increase levels of anti-inflammatory biomarkers such as IL-10 and adiponectin [14,15,16,17,18].

High consumption of vegetable polyphenols in the MD may have the potential to increase the capacity to neutralize free radicals, enhance physical performance, and aid in recovery from muscle micro-damage [19,20]. Polyphenols may accelerate recovery by improving antioxidant status and suppressing inflammation. However, evidence in the literature regarding their effects on exercise-induced muscle damage ranges from moderate to very low [21]. Polyphenols; other plant-derived substances; probiotics; and vitamins A, C, and D, as well as carbohydrates abundantly present in the MD, support the immune system (including immune cells and chemotaxis) and may aid in immune recovery for athletes [22,23]. Additionally, the high nitric oxide (NO) content in the vegetable-rich MD, when consumed before exertion, contributes to improvements in cardiovascular and respiratory parameters, physical endurance, high-intensity intermittent exercise performance, muscle power, and sprinting ability [24,25].

Olive oil in the MD is rich in bioactive compounds such as monounsaturated fats (MUFA), phenolic compounds, polyphenols, and vitamins and possesses anti-inflammatory, antioxidant, and microbiota-regulating properties that may support athlete recovery [26]. Replacing saturated fatty acids with MUFA can reduce metabolic stress and prevent the expression of inflammatory genes in various tissues. Additionally, the phenolic components and other phytochemicals in olive oil have positive effects on metabolic parameters, including plasma lipoproteins, oxidative damage, inflammatory markers, pro-thrombotic markers, platelet function, and antimicrobial activity [27]. Fish oil consumption in the MD is rich in omega 3 fatty acids Docosahexaenoic acid (DHA) and eicosapentaenoic acid (EPA) and can improve sporting performance, enhance recovery, and prevent illness/injury, by improving the immune system and decreasing oxidative stress [28].

A recent systematic review (SR) and meta-analysis [2] on the relationships between the MD and components of physical fitness (PF) including cardiorespiratory fitness (CRF), motor fitness (MF) (i.e., motor skills such as agility or speed), and musculoskeletal fitness (MSF) (i.e., muscle abilities such as flexibility or muscular strength) show a significant positive association between high (compared with low) MD adherence and CRF, MSF, and overall PF in adults of all ages. Of the 30 selected studies, 3 focused on young adults, 5 on middle-aged adults, and 22 on older adults. The authors highlighted the need for more research on young and middle-aged populations.

According to Griffiths et al. (2022), despite its potential health and performance benefits for competitive athletes, the application of the MD in a sporting context has received very little attention to date [29]. Those authors present compelling arguments about the potential of the MD to enhance sports performance, drawing on findings from numerous scientific studies. However, they identify only a single study directly focused on this topic, which involves recreationally active participants.

In their review on the dietary impact on athletic performance, Kaufman et al. (2023) [30], regarding the MD, refer to two studies involving athletes that provide some evidence for improved physical performance. However, they note that these studies lacked a control group [30].

In their systematic review on the impact of the MD on athletic performance, muscle strength, body composition, and antioxidant markers, Bianchi et al. (2024) [31] selected five studies that assessed physical performance. They conclude that while the MD appears to be a promising dietary strategy for enhancing athletic performance and overall health, more rigorous research is needed to clarify its effects across diverse athletic populations [31].

According to the reviewers, the association between the MD and physical performance in athletes remains underexplored, with methodological gaps and contextual differences across studies. We propose a scoping review to identify these gaps in the existing literature, summarize the key findings, and highlight the implications and future research needs to enhance understanding in this area.

## 2. Materials and Methods

This study was conducted following the guidelines provided by Cochrane [32] and the instructions outlined by the Preferred Reporting Items for Systemic Review and Meta-Analysis (PRISMA) [33]. The protocol was registered at Open Science Framework: doi.org/10.17605/OSF.IO/3CJM5.

### 2.1. Search Strategy

The research was conducted across four databases—PubMed, Scopus, Scielo, and Google Scholar—using the following terms and their combinations as the search strategy: “mediterranean diet”, “performance”, “athlete”, and “sport”. These keywords were defined after reviewing scientific abstracts that addressed the influence of diets on athletic performance. From these abstracts, the most frequently reported terms in the titles or keywords were those included in the current search strategy. Subsequently, the references were exported to specific software (EndNoteTMX9, Clarivate Analytics, Philadelphia, PA, USA) [34]. The article search and data extraction were carried out between 17 December 2023 and 30 October 2024.

### 2.2. Eligibility Criteria

Inclusion Criteria:

The inclusion criteria included studies published in English in peer-reviewed journals that included healthy athletes, regardless of competitive level, age, or sex. Participants had to be evaluated for their adherence to the MD and assessed for their physical performance. No restrictions were placed on the experimental design or the time period during which articles were searched.

Exclusion Criteria:

The exclusion criteria included articles describing physically active recreational participants; studies that did not include parameters related to the MD or physical performance assessment; studies involving participants with associated pathologies; animal studies; and review articles or meta-analyses, commentaries, editorials, or conference abstracts.

### 2.3. Study Selection

After removing duplicates, two authors (A.R. and A.M.) performed the selection of articles based on the title and abstract. For the articles selected by title and abstract, the full text was extracted, and compliance with the previously mentioned eligibility criteria was verified. In both selection processes (by title and abstract or by full text), in cases of inconsistent decisions, a third independent author (D.M.) participated in the selection process, allowing for a consensus decision among the authors.

### 2.4. Data Extraction

The following data were extracted from the studies deemed eligible by full text, organizing them in a Microsoft Excel sheet. The following information was extracted from the studies: journal of publication, name of the first author and year of publication, country, study design, sample size, participant characteristics (age, sex, sport practiced, level of practice), adherence to MD assessment, the main results with a focus on athletic performance, limitations, and practical applications.

### 2.5. Quality Assessment

In this review, three different tools were used, one for each type of study included. We used the Quality Assessment Tool for Observational Cohort and Cross-Sectional Studies [35], the Quality Assessment Tool for Before-After Studies [36] for studies with two performance evaluation points (before and after), and the Quality Assessment of Controlled Intervention Studies [37] for randomized longitudinal studies.

Each question of the tool could be answered as yes (no risk of bias), no (risk of bias), cannot determine, not applicable, or not reported. A study was classified as good if it answers yes for 60–100% of the tool items, fair for 50–59%, or poor for 0–49%.

## 3. Results

### 3.1. Identification of Relevant Research

**Figure 1:**
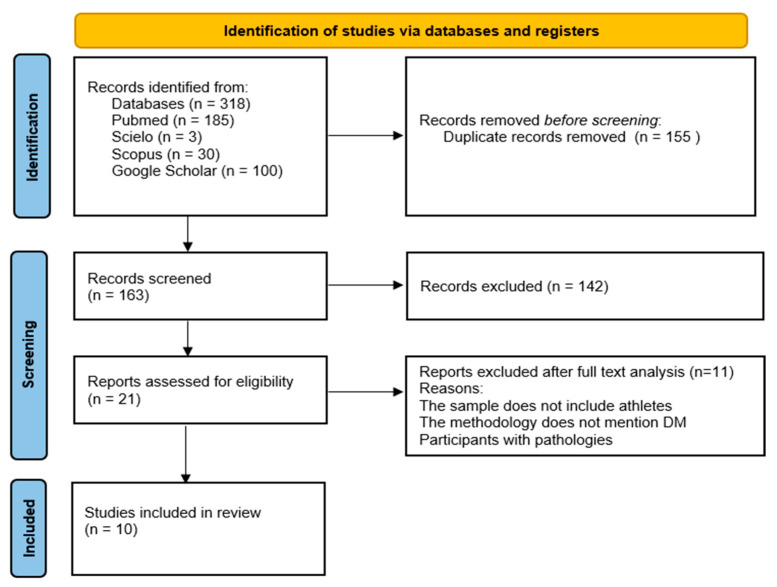
PRISMA flowchart of the selection process.

The research across the four databases identified 318 scientific articles. Subsequently, 155 duplicates were removed. Of the 163 articles initially selected, 142 were excluded based on the title and abstract. Thus, 21 articles were analyzed in full text, confirming or not the eligibility criteria. Of these, 11 did not meet the eligibility criteria and were excluded for the following reasons: the sample did not include athletes; the diet used did not correspond to the MD; and finally, studies were found in which the participants had an associated pathology. In this review, 10 studies were considered eligible [38,39,40,41,42,43,44,45,46,47]. The identification of relevant research is summarized in Figure 1.

### 3.2. Study Characteristics and Results

Table 1 summarizes the selected articles’ references, the countries where the studies were conducted, the sample characteristics, the study details, the variables evaluated, and the main results. As expected, most of the studies were conducted in countries with MD traditions. Five studies were carried out in Spain, three in Italy, one in Turkey, and one in Greece. Upon reviewing the studies, a considerable variability in sample characteristics can be identified. Five studies exclusively focused on adults [38,39,44,46,47], and two focused on young participants [40,45]. Additionally, three studies included participants from different age groups [41,42,43]. Four studies included participants with varying levels of sports practice: one study focused on professional athletes [39], two on national-level team athletes [42,43], two on federal-level athletes [40,41], and one on individuals with moderate activity levels [38].

Half of the types of studies found in this review were cross-sectional or transversal studies [41,42,43,45,47]. To assess adherence to the MD, the Mediterranean Diet Quality Index in Children and Adolescents or KIDMED was used in four studies of young participants, and the Meddiet score was used for adults. The remaining studies were longitudinal and proposed an MD plan.

Regarding the physical test analyzed, a great variety of tests were used among the studies and could be characterized as strength tests (handgrip dynamometry, vertical jump, squat jump, countermovement jump, medicine ball throw, squat lift, elbow flexor, and knee extension maximal voluntary contraction), aerobic tests (shuttle run, yo-yo, and maximal oxygen consumption), speed and agility tests (T-half test and cycling speed), anaerobic tests (Wingate and 30 s jump test), and modality-specific tests.

Regardless of the study design, six manuscripts did not observe any effect of the MD on performance [38,39,41,42,43,47]. However, in four studies [40,44,45,46], the MD had a significant impact on athletic performance. Last, the results were not consistent between male and female participants or between age groups.

### 3.3. Risk-of-Bias Assessment

The methodological quality of the included studies is presented in Table 2, Table 3 and Table 4. In the overall analysis, with the exception of two studies classified as poor, the risk of bias was rated as fair in three studies, while the remaining studies were rated as good. Among the cross-sectional studies, the sample power was not calculated, while only one longitudinal study provided this information. Of the five longitudinal studies, four used control groups, and three implemented randomizations for group assignments.

## 4. Discussion

This study aimed to assess the impact of the MD on athletic performance through a scoping review, building on inconclusive findings from previous reviews to summarize key insights and identify gaps for future research.

We identified only 10 studies linking the MD to performance, confirming the scarcity of research in this area, as highlighted in other literature reviews [29,30,31]. Our quality assessment of the selected studies indicated that four were of good quality, four were of fair quality, and two were of poor quality, with 80% meeting the criteria to minimize the risk of bias. These findings aligned with Bianca et al. (2024), whose systematic review [31], using a different evaluation tool (RoB-2), found a moderate risk of bias in four common longitudinal studies.

Despite an apparently adequate quality, only 40% of our selected studies found a significant impact of the MD on athletes’ physical performance, contrasting with the strong theoretical basis supporting the MD’s potential benefits for athletic performance presented by Griffiths et al. (2022) [29]. Drawing on extensive research, those authors suggest that the Mediterranean diet (MD), rich in ergogenic compounds like nitrate-rich vegetables, omega-3 fatty acids, a variety of different fruits, certain types of nuts, and antioxidant sources such as olive oil or moderate red wine consumption, could effectively reduce exercise-induced oxidative stress and support athletic performance without relying on supplements. As a cost-effective and enjoyable dietary approach, the MD may help athletes meet sports nutrition goals while gaining added health and performance benefits [29].

As mentioned in the previous review of the literature [29,30,31], the participants’ athletic level, sport type, and age were inconsistent, making it challenging to draw firm conclusions. Our analysis revealed considerable variability in sample characteristics across the selected studies. Five studies focused solely on adults, two focused on younger participants, and three included mixed-age groups. Additionally, the level of sports practice varied: four studies included athletes at different practice levels, one focused on professional athletes, two focused on national-level team athletes, two focused on federal-level athletes, and one focused on moderately active individuals. Regarding sex, two studies presented results on adult females, and one presented results on young females; in contrast, four studies focused on adult males, another four focused on young males, and two presented mixed-sex results. This variability in participant demographics and athletic levels limited the generalizability of the findings, underscoring the need for more standardized, diverse sampling to clarify the MD’s impact on athletic performance across demographics.

Focusing on the selected longitudinal studies, one study [40] involving young athletes reported promising results after 15 days of the MD, showing significant improvements in handgrip dynamometry, vertical jump, and shuttle run performance, suggesting enhanced strength and aerobic endurance. These improvements were also associated with a reduced perception of fatigue. However, the lack of a control group and the significant increase in the subjects’ stature during the study, likely due to natural growth, compromised the validity of these findings.

In another study [44], adult CrossFit athletes of both sexes showed significant improvements in the Wingate anaerobic test, vertical jump, and CrossFit-specific performance tests after 8 weeks of the MD compared with a control group, indicating the potential of the MD to enhance strength, lactic anaerobic endurance, and the mixed combination of aerobic–anaerobic. Supporting these findings, a study on adult kickboxers and half-marathoners [46] reported significant improvements in countermovement jump, 15 s jump, and maximal oxygen consumption in half-marathoners following 12 weeks of MD supplementation, while their control group showed no improvement and a significant decrease in squat jump, indicating the potential of the MD to enhance strength, anaerobic endurance, and aerobic endurance. Kickboxers also showed improvements in squat strength and countermovement jump over the same period; however, these gains did not surpass those of the control group. It is important to note that the authors did not conduct any inferential statistics to compare the control and experimental groups, limiting their analysis to a subjective comparison.

Additionally, two studies [38,39], one involving professional adult female athletes and the other a moderately active mixed-sex group of adult athletes, found no improvement in physical performance following 6- and 12-week MD interventions, despite employing well-designed methodologies. In the study involving professional athletes [39], the lack of effect may be attributed to insufficient calorie consumption among the participants.

For optimal sports performance in highly trained athletes, adequate carbohydrate (CHO) and water (W) consumption is essential to sustain their training load throughout the week. The daily recommendations suggest 6 to 10 g of CHO per kg of body mass [48,49,50]. Fluid intake should follow general guidelines of 30 mL per kg of body mass for the average person, with additional amounts to meet training demands [51,52], which can increase to 45 mL/kg/day or more, depending on individual and sport-specific needs [53,54]. Despite these important recommendations for athletic performance, only two of the selected longitudinal studies reported their diet log results, while three studies kept diet logs but did not present their findings. Although the ACSM [55] warns of the risk of misinterpretation when using the percentage of total caloric intake for CHO (%CHO) instead of grams of CHO per kg of body mass, none of the studies reported CHO intake in g/kg/day. Three studies used the percentage of total caloric intake, one reported the total grams consumed along with the percentage, and one did not report CHO consumption at all. None of the studies presented results or information regarding fluid consumption.

Based on the available information in the studies, such as body weight; total caloric intake; or extrapolations from resting metabolic rates, grams, or percentage of CHO consumed, in the MD groups we estimated average CHO intakes of 2.27, 5.7, and 8.1 g/kg/day across three different studies. These substantial differences in CHO intake likely influenced the study outcomes.

Additionally, all five longitudinal studies demonstrated changes in body composition with the MD, but only one reported a correlation with physical performance outcomes, highlighting a limited exploration of statistical possibilities that also impacted comparisons between the experimental and control groups. Although it is well known that the conditions under which physical tests are conducted can influence results [56,57,58], notably, only two studies addressed the equivalence of rest and nutritional conditions before and during testing. Furthermore, none of the studies mentioned a familiarization period for participants with the tests or the timing and order of test administration across different points in the study. There is also a lack of information regarding training schedules, complicating the classification of the athletes’ skill levels, especially in studies with mixed-level participants. Moreover, supplementation control and, in female participants, the regulation of menstrual cycles were not consistently considered across these studies.

Only one study calculated the sample size, estimating that 13 participants would be sufficient to detect differences in physical performance between trials; however, it did not find any significant influence of the MD. Another study, which also did not yield significant results, included 7 participants, while the study that showed some improvement with the MD included groups of 10, 15, and 20 participants.

While the MD showed potential benefits in strength, endurance, and fatigue perception, many studies lacked control groups, statistical rigor, and control over key variables, such as diet, hydration, and participant familiarization with tests. These gaps, along with insufficient reporting on CHO and fluid intake, compromised the reliability of the results and suggested a need for more stringent methodological standards in future research. Despite these gaps, among the five selected studies where the MD was implemented for 2 to 12 weeks, only one did not show an improvement in physical performance. Another study showed improvement but did not differ significantly from other groups, while the remaining three demonstrated clear improvements. These findings suggest that effective implementation of the MD has potential as an option to enhance athletic performance.

In the five selected cross-sectional studies, only one [45] reported significantly better results in the shuttle run (for the entire group) and in handgrip tests (only for males) among participants with high adherence to the MD compared with those with medium and low adherence. This study had the largest sample size, including 1998 participants (875 males and 323 females), while the other studies involved sample sizes ranging from 7 to 50 participants, which may have been insufficient to achieve the statistical power needed to detect significant results [59]. None of these cross-sectional studies specified the conditions of the physical tests, such as exercise order, rest periods, or nutritional aspects; only one mentioned a familiarization period for the tests. MD adherence was measured only once and was not cross-referenced with a diet log (e.g., diary or food frequency questionnaire). Three out of five studies reported correlations between performance and body composition outcomes.

This analysis points out that only one study among the five had a robust sample size and reported significant performance improvements associated with high MD adherence. The smaller sample sizes in the other studies likely limited their statistical power. Additionally, a lack of standardized testing conditions and limited dietary tracking across studies further complicates the interpretation of the MD’s effects on physical performance.

## 5. Conclusions

We found only 10 studies linking the MD to performance, highlighting a scarcity of research in this area. The evidence of MD efficacity is limited; only 40% of the reviewed studies showed a significant impact of the MD on athletic performance, which contrasts with the theoretical support for the MD’s benefits. Additionally, it is debatable whether the methodologies of these few studies were robust enough to support their conclusions.

There was considerable heterogeneity in participant demographics, athletic level, and testing conditions across studies. Such variability, including differences in age, sex, and sports level, limits the generalizability of the findings and prevents a clear understanding of the MD’s effects on athletic performance.

Methodological gaps included the following: Few studies controlled for essential factors that influenced physical performance outcomes, such as consistent testing conditions (exercise order, rest, and nutrition) and proper familiarization periods. Additionally, inconsistent diet monitoring and lack of control groups further reduced the reliability of the results.

Carbohydrate and fluid intake, crucial for interpreting athletic performance, were poorly reported. Only a few studies included detailed diet logs, and none provided information on fluid consumption, potentially skewing results and limiting insights into how these variables might interact with the MD.

## 6. Practical Implications for Future Studies

Future longitudinal studies should aim to do the following:

Aim to create more homogeneous participant demographics by controlling for athletic level, age, sport type, and sex. Detailed information on participants’ training schedules should be provided to accurately assess their athletic level, ensuring the application of appropriate experimental treatments and avoiding the inclusion of mixed-level groups in statistical analyses. The same precautions should be taken for age, sport type, and sex. This approach will enable clearer insights into how the MD affects different populations of athletes.

Provide larger sample sizes of a minimum of 15 to 20 participant in the MD group to enhance the statistical power and increase the likelihood of detecting significant differences in athletic performance associated with the MD.

Include control groups and standardized testing conditions, incorporating familiarization periods for participants and clear protocols for exercise order and rest periods. Additionally, ensure consistent nutritional conditions between test sessions to enhance the reliability of the results.

Provide comprehensive dietary assessments to rigorously track dietary intake, including detailed evaluations of carbohydrate intake (in g/kg/day), fluid consumption, and ergogenic supplementation. Additionally, relate nutritional and athletic performance outcomes to changes in body composition. This will help account for potential confounding variables that may influence performance outcomes and verify consistency in testing conditions.

Future cross-sectional studies should aim to do the following:

Aim to include larger and more diverse samples of participants to improve the generalizability of findings and better understand the MD’s impact across various athlete demographics, avoiding mixed-level, sex, age, or sport type group treatments.

Implement standardized testing conditions to control for variables such as exercise order, rest periods, and nutritional patterns, increasing the reliability of the results and facilitating more accurate comparisons across studies.

Enhance the measurement of MD adherence by using multiple assessment methods, such as diet logs or food frequency questionnaires, to accurately capture participants’ consumption patterns.

Provide insights into changes in performance over time in relation to MD adherence, allowing for a better understanding of causal relationships.

Investigate the mechanisms through which the MD may influence performance outcomes, with specific reference to the variables studied.

## Figures and Tables

**Table 1:** Characteristics of the studies included and main results.

Reference/Sample	Study Characteristics	Main Results
Perna et al. (2024) [38], Italy. 13 Moderately active strength-trained athletes (9 males and 4 females), 25.8 ± 4.2 y	Crossover randomized control study, 8 weeks + 6 weeks washout + 8 weeksDescription of training level, compliance, and rest prior to testing SJ, CMJ, HG, elbow flexor, and knee extension MVC	No significant improvement of physical performance with the MD (55–60% CHO). And decrease in strength and midarm circumference with the MD (40–45% CHO).
Miralles-Amorós et al. (2023) [39], Spain. Female professional handball. 7 MD, 7 C, 7 Antiox, 22.0 ± 4.0 y	Randomized controlled trial: 12 weeks Training level description Diet log (results presented) Nutritional control and rest prior to testing CMJ, Abalakov jump test, and HG	Improvements in CMJ were observed in the three groups at the end of the 12 weeks. However, the time and group effects were not significant in any of the groups.
Helvaci et al. (2023) [40], Turkey. 15 Male adolescent ski-running elite, 14.9 ± 1.3 y	Longitudinal study: 15 days of MD intervention pre- and post- test, without control group Training level description Diet log (results presented) and control of compliance No specified test conditions HG, VJ, and SR with perception of effort and lactate	The application of the MD allowed for improvements in VJ, HG, and SR performance and reduced the perception of fatigue during the shuttle run.
Garcia et al. (2022) [41], Spain. 145 male handball federate, from infants 13.4 ± 0.4 y to senior 20.1 ± 2.8 y	Cross-sectional study KIDMED, no diet log control Familiarization to tests, no specified test conditions Yo-yo test, SJ, CMJ, linear speed, medicine ball throw, and T-half test protocol	Although significant age-related differences were observed in physical condition variables, no similar trend was found for adherence levels to the MD.
Martínez-Rodriguez et al. (2021a) [42], Spain. Female beach handball national team, 18 junior and 15 senior	Cross-sectional study Training level description KIDMED, no diet log control No specified test rest and nutritional conditions Yo-yo test, Abalakov jump test, and HG	No positive effects of MD adherence on physical performance tests were observed. In the senior group, negative correlation was found with the Abalakov jump test.
Martínez-Rodriguez et al. (2021b) [43], Spain. National team beach handball, 38 junior/senior of both sexes	Cross-sectional study Training level description KIDMED, no diet log control No specified test rest and nutritional conditions CMJ and HG	No positive effects of MD adherence on physical performance tests were observed. In the junior female group, negative correlation was found with HG.
Ficarra et al. (2020) [44], Italy. Adults, CrossFit of varying levels. MD: 6 males and 4 females, C: 7 males and 4 females, 35.6 ± 8.4 y	Longitudinal study: 8-week MD intervention. Pre- and post-test, with control group Training and compliance description Diet log (no results), no level discrimination No specified test conditions VJ, CMJ, 30 s jump test, Wingate anaerobic test, push-ups and chin-ups, and CrossFit tests	The MD showed improved Wingate anaerobic test, VJ, and CrossFit tests, whereas the control group did not. Both groups improve in push-up and chin-up performance.
Carrasco et al. (2020) [45], Spain, 1998. Young sports athletes of varying levels, both sexes	Cross-sectional study No training level and test condition descriptions KIDMED, no diet log SR, HG, CMJ, and respiratory capacity (RC)	MD adherence positively influenced SR results in the overall population and HG and respiratory capacity among males.
Soldati et al. (2019) [46], Italy. Male adult athletes of varying levels, 20 kickboxer, 18–38 y, 20 half-marathon	Randomized controlled trial: 12 weeks Description of compliance No specified test rest and nutritional conditions Squat and bench flat strength, SJ, CMJ, 15 s jump test, and VO2max	With the MD, in kickboxers squat strength and CMJ and in runners VO2max, CMJ, and 15 s jump test increased significantly, while in runners, the control SJ decreased.
Papadopoulou et al. (2017) [47], Greece. 50 varying level male cyclists, 15–50 y	Cross-sectional study Training description, no level discrimination Meddiet score, no diet log control No specified test rest and nutritional conditions Cycling endurance and speed test	MD adherence level showed no significant impact on endurance and speed test results.

y = years; w = week; RC = reduced carbohydrates; MD = Mediterranean diet; VJ = vertical jump; HG = hand grip; MVC = maximum voluntary contraction; SR = shuttle run; SJ = squat jump; CMJ = counter movement jump; VO2max = maximal oxygen consumption; C = control group.

**Table 2:** Quality assessment for cross-sectional studies.

Authors/Questions	1	2	3	4	5	6	7	8	9	10	11	12	13	14	Classif.
García et al. (2022) [41]	1	1	1	1	2	2	2	1	1	2	1	3	1	1	G
Martinéz et al. (2021a) [42]	1	1	1	1	2	2	2	1	2	2	2	3	1	2	P
Martinéz et al. (2021b) [43]	1	1	1	1	2	2	2	1	1	2	1	3	1	2	F
Carrasco et al. (2000) [45]	1	2	1	1	2	2	2	1	1	2	1	3	1	2	F
Papadopoulou et al. (2017) [47]	1	2	1	1	2	2	2	1	1	2	2	3	1	2	P

Classif. = global classification of the study; G = good; F = fair; P = poor; 1 = yes; 2 = no; 3 = not applicable.

**Table 3:** Quality assessment for before–after studies.

Authors/Questions	1	2	3	4	5	6	7	8	9	10	11	12	Classif.
Helvaci et al. (2023) [40]	1	1	1	1	2	1	1	3	1	1	2	1	G

Classif. = global classification of the study; G = good; 1 = yes; 2 = no; 3 = not applicable.

**Table 4:** Quality assessment of controlled intervention studies.

Authors/Questions	1	2	3	4	5	6	7	8	9	10	11	12	13	14	Classif.
Perna et al. (2024) [38]	1	1	1	3	3	1	1	1	1	2	1	1	1	1	G
Miralles-Amorós et al. (2023) [39]	1	1	1	3	3	1	1	1	1	1	1	2	1	1	G
Ficarra et al. (2020) [44]	2	1	2	3	3	1	2	1	1	2	1	2	1	1	F
Soldati et al. (2019) [46]	2	1	2	3	3	1	1	1	1	2	1	2	1	1	F

Classif. = global classification of the study; G = good; F = fair; 1 = yes; 2 = no; 3 = not applicable.
